# Opinion Review of Socioeconomic Impact of COVID-2019 on Women's Health

**DOI:** 10.3389/fgwh.2021.647421

**Published:** 2021-05-13

**Authors:** Victory U. Salami, Stanley I. R. Okoduwa, Aimee O. Chris, Susannah I. Ayilara, Ugochi J. Okoduwa

**Affiliations:** ^1^Scientific and Industrial Research Department, National Research Institute for Chemical Technology, Zaria, Nigeria; ^2^Department of Biochemistry, Babcock University, Ilishan-Remo, Nigeria; ^3^Directorate of Research and Development, Nigerian Institute of Leather and Science Technology, Zaria, Nigeria; ^4^Industrial and Environmental Pollution Department, National Research Institute for Chemical Technology, Zaria, Nigeria

**Keywords:** SARS-CoV2, COVID-19, women's health, socio-economic status, coronavirus

## Abstract

The global battle to survive the onslaughts of the Coronavirus Disease 2019 (COVID-19) started in December 2019 and continues today. Women and girls have borne the brunt of the hardship resulting from the health crises. This paper examined the effects of COVID-19 on women. Socioeconomic factors resulting from the pandemic, especially in relation to women's health, were discussed after studying published articles. They include gender specificity and COVID-19, the economic toll of COVID-19 on women, pregnancy and COVID-19, gender-based violence due to COVID-19, and health-care impacts of COVID-19. Making up the majority in the healthcare workforce, women were at higher risk of infection with COVID-19 due to their exposure as caregivers to infected patients. The pandemic took its toll on them as part of the greater population in the informal sector of the economy due to the lockdown directive, as many experienced severe monetary shortages and job losses. Pregnant women infected with COVID-19 were prone to severe diseases, maternal complications, and death due to their weakened immunity and exposure during clinical procedures. Gender-based violence was observed to have increased across the globe for women. The results of this review strongly indicate that women are disproportionately affected by the ongoing COVID-19 health crisis. This review will help health-care professionals and policymakers arrive at properly-thought-through decisions to better manage health crises. Governments and all key players should address the challenge by devising effective policies with a gendered view.

## Introduction

Severe Acute Respiratory Syndrome Coronavirus-2 (SARS-CoV2), also called COVID-19, is a new coronavirus infection identified in December 2019 originating from Wuhan, China (1). Since its outbreak, it has spread to more than 220 countries in the world with rising cases of fatality. Some regions have overcome the third wave of the crisis and are facing the start of a fourth (2). As of February 1, 2021, 13:00 GMT, the weekly update of the World Health Organization reports that there have been over 100 million confirmed cases with more than two million confirmed deaths globally (3). This disease has initiated a fast-growing global crisis that has taken researchers and policymakers without notice, thus leaving them scrambling to collect and analyze data so that its true impact on affected countries can be grasped (4).

As the world begins vaccination to manage the COVID-19 pandemic, preventive measures have been advocated, including physical distancing. The current pandemic and the imposed physical/social distancing policy are emphasizing health inequalities based on gender, socioeconomic status, and race (5–9), thereby highlighting the discrimination of other marginalized groups, such as internally displaced people, those in extreme poverty, people with disabilities, migrants, and the refugees whose majority are women and children (10). The gendered effect of COVID-19 on women presents a huge challenge that cannot be ignored. Deliberations about the health effects of COVID-19 should include unique conditions that make women more at risk (11).

In this review, research findings from publications retrieved online were used to highlight some health and socioeconomic challenges due to the COVID-19 pandemic that are particular to women. The discussion was based on COVID-19 in relation to five key factors: (1) gender specificity (is COVID-19 neutral to men and women?), (2) economic toll (has COVID-19 affected the economic life of women?), (3) pregnancy (does COVID-19 affect pregnant women more than non-pregnant women?), (4) gender-based violence (has COVID-19 affected the rate of gender-based violence?), and (5) health care (how have women fared in accessing sexual and reproductive health care during the COVID-19 pandemic?). If the items discussed in this paper are properly taken into consideration, the likelihood of future health crises would be better managed.

## Methods

The study employed a literature search of majorly published journal articles written in English from electronic databases such as ScienceDirect, PubMed, and Google Scholar. Information from the World Health Organization and the United Nations Women databases was also accessed. The literature search was performed by all authors listed on the paper between July 15, 2020, and February 3, 2021. Articles were searched using the following keywords: women; COVID-19; impacts of COVID-19 or effects of COVID-19; economic impacts of COVID-19; social effects of COVID-19; health impacts of COVID-19; COVID-19 pandemic; SARS Cov-2; and Coronavirus.

### Population

Females in this article refer to women and girls. The ages of the females who participated in the studies included in this review ranged from 13 years old and above.

### Selection Criteria

Reports and full-text articles related to the health, social, and economic impacts of COVID-19 on women were selected. The study excluded reports and articles that excluded the impacts/effects of COVID-19 on women, articles not written in English, as well as information derived from blogs and other unverifiable sources.

## Result and Discussion

A total of 1,518 records were identified based on selection criteria of key search words: 1,006 from the databases searched and 512 from other sources. After screening to remove articles that did not capture the context of interest, duplicates, and articles not written in the English Language, a total of 291 articles were selected. Of the 291 selected articles, 266 were not suitable because they were misleading in context and were thus excluded. The remaining 25 selected articles were then included for analysis.

### Gender Specificity and COVID-19

Gender is a path upon which the COVID-19 pandemic is widening health inequalities (11). Years before this pandemic, women typically reported more physically unhealthy days than men even though they used more preventive care services (12). Women have poorer results for widespread diseases such as myocardial infarction (13), asthma (12), and diabetes (14). This health discrimination is aggravated for women of low socioeconomic status, older age, physical disability, lower education, residence in rural geographic locations, and those who are not white (11). In a recent web-based survey of 780,961 participants from 183 countries, a major risk association for infection with COVID-19 was observed for people who were of the female gender, aged above 60 years, and those who had preexisting disease conditions, such as heart disease, kidney disease, diabetes, and liver disease (15). Women are at greater risk of mental health challenges and exhibit more psychological distress than men (16). Based on the available literature, COVID-19 health crises will likely distress women mentally to a higher degree (16). In a study on mood, empathy, and sleep quality during the isolation period due to COVID-19 in Canada, the authors discovered that women suffered more due to trauma symptoms, depression, and increased anxiety (17). Many health-care staff members could develop posttraumatic stress disorder due to burnout, depression, and anxiety during and after the pandemic, and this is another factor supporting the weightier impact of the pandemic on women (18).

COVID-19 does not discriminate against gender (male or female), health status (healthy or immuno-compromised), or age (although infection in children is less common, most affected adults are between 25 and 89 years old) (19, 20). Initial reports indicated that men were at greater risk of severe disease and death from COVID-19 infection compared with women (7, 21, 22). Researchers warned that those early investigations were to be treated with caution because they are incomplete and inconsistent across countries (23, 24). Current statistics of sex-disintegrated data (which is presently incomplete) show that more women test positive for COVID-19 compared to men (25). Recognizing the direct and indirect effects of the disease on women will assist in providing effective responses for similar health crises in the future (26).

Across the globe, about 70% of health-care workers (doctors, nurses, midwives, community health workers, cleaners, caterers, and launderers) are women, of which 80% are nurses in most countries. These women are particularly at risk of infection through contact with sick patients (23). Factors associated with fatalities of patients with COVID-19 include older age, obesity, diabetes mellitus, hypertension, and cardiovascular diseases (27).

### The Economic Toll of COVID-19 on Women

Although physical distancing slows down the transmission of COVID-19, the advantage of this measure needs to be balanced with its effects on the informal sector of the economy (8). The implementation of the lockdown measure in developing countries (particularly in Africa where poverty, weak health-care systems, and overcrowding exists) has taken its toll on all (especially women) (8). Many women in the non-formal sector have limited access to social security, and, as such, widespread job losses will have a long-term impact on their economic independence and security (4).

An estimated 740 million women are employed in the informal sector of the economy, which includes tourism, hospitality, and retail. In developing countries, this workforce constitutes more than two-thirds of women. To make a living, they rely on open public space, which is at present being limited to curb the spread of the virus. Therefore, severe monetary shortfalls are very much felt by these women who earn less, save less, and hold more insecure jobs compared to their male counterparts (7, 28).

COVID-19 and the restrictions put in place to curtail its spread have interrupted markets and businesses and many have lost their means of living (29). In April 2020, The International Labour Organization (ILO) projected that lockdown will distress about 3 billion employees (81% of the global labor force) and that the COVID-19 pandemic could cost between 5 million and 25 million jobs (7). Recently, the ILO posits that estimations of revenue losses from labor show a global decline of 10.7% during the first three quarters of 2020 compared with the corresponding period in 2019, which amounts to 3.5 trillion United States dollars (5.5% global gross domestic product) (30). The International Monetary Fund projected a major shrinkage of global output in 2020 (31).

According to Clare Wenham, assistant professor of Global Health Policy, London School of Economics and Political Science, the economic toll the COVID-19 pandemic has on women is an indirect consequence, coming not from being infected but from being affected (28). Despite a major limitation from the fact that not all countries provide sex-disaggregated data, a clear trend of the pandemic's indirect effect on women has appeared (28). Women have less access to social protection, and most single-parent households are women. The capability of women to absorb economic shocks is less than that of men (28). The Institute for Fiscal Studies found that women in the United Kingdom were 1.5 times more likely than men to have either quit their job or lost it during the lockdown (28). As countries experienced lockdown, jobs were lost with disastrous consequences on the holders.

The care burden on women has increased due to the imposed lockdown—care for the sick and elderly, care for children forced to stay at home because their schools have been ordered to stop, and care for other family members who are all locked down at home. Consequently, during the pandemic, women are the leading participators in an unnoticed economy as unpaid caregivers in the family. This unnoticed economy has actual consequences on the economy and lives of women. It is strongly recommended that governments offer inclusive social security for all caregivers to lessen the burden on unpaid care workers (7, 32).

### Pregnancy and COVID-19

Several studies have been conducted, and they unanimously agree that pregnant women could be more prone to contracting COVID-19 due to their weakened immune systems (33–35). Pregnant women who are infected with the COVID-19 virus are at more risk of serious diseases and greater risk of being hospitalized, subjected to mechanical ventilation, and being admitted to the intensive care unit compared with non-pregnant women with COVID-19 (36). This observed susceptibility in pregnant women is probably partly due to exposure risk from clinical settings and practices as well as the physiological changes that occur in pregnancy (11). In infected pregnant women, there is no proof of vertical transmission of the virus to the unborn child; however, there are higher incidences of preterm deliveries (37, 38). Furthermore, several authors reported severe maternal complications related to pregnant women with COVID-19 (39–41) and even deaths (42, 43). After systematically reviewing 19 studies of pregnant women with severe acute respiratory syndrome (SARS), middle east respiratory syndrome (MERS), or COVID-19, Di-Mascio and his fellow researchers revealed that infected pregnant women had more cases of Cesarean delivery, preeclampsia, preterm birth, and maternal death (34, 35, 44, 45).

Data on COVID-19 infections in pregnancy have been mainly from North America and Europe. Early confirmation from low and middle-income countries such as Nepal and Uganda shows that though the rate of infection is not high among the population, imposed restrictions due to the pandemic are distressing maternal-child outcomes, showing a severe drop in maternal facility births (up to 50% in Nepal) and amplified neonatal and maternal morbidity (46, 47).

### Gender-Based Violence Due to COVID-19

Crises (natural disasters, war, or epidemics) accentuate incidences of gender-based violence against women (48, 49). This was the trend during the Ebola (2014) and Zika (2016) outbreaks (50). The existing challenge of physical abuse against women will expectedly worsen with COVID-19. Developing data indicate that since the pandemic broke out, domestic and sexual violence have risen in many regions and will likely escalate (51–53). As health, security, and financial challenges cause tension and strain in families, confinement because of the lockdown directive intensifies this pressure and makes people (particularly men, angered and frustrated by their lack of money) more prone to domestic violence toward their female partners (54–56). Some of the risk factors underlying gender-based violence include the narrowness of accommodation due to overcrowding, social isolation, fear of dying, low income, reduced access to services, decreased peer support, increased consumption of addictive substances, and male aggression (7, 51, 57–60). Since the implementation of the lockdown by governments around the world, many countries have observed an upsurge in cases of gender-based violence. Government authorities, civil society groups, and women's rights activists have indicated rising cases of gender-based violence across the globe (51, 52). The Refuge website has equally reported that calls about gender-based violence have increased by 150% (61). Intimate partner violence was reported to have increased by 30% in France and Cyprus, 33% in Singapore, and 25% in Argentina (53). Different states in the US also reported an intimate partner violence increase of 21–35% (62). Increased gender-based violence and demand for emergency shelter were also reported in Spain, Canada, Germany, and UK (53). In Nigeria, with the initial lockdown of three major states—Lagos, Ogun, and the Federal Capital Territory—throughout April 2020, gender-based violence significantly increased by 56% (49). During the first 2 weeks of lockdown, gender-based violence cases rose from 346 to 794 (40).

Health-care services are currently inundated with COVID-19 cases. In places where basic vital services are sustained, a breakdown in harmonized response from important sectors such as health, justice, police, and social services coupled with the physical/social distancing measure implies that sectors will be limited to provide satisfactory care to women who are suffering violence (7). That is to say, though shelters are available for women experiencing violence to take refuge, the lockdown order has compelled bodies such as judicial courts (in which the legal advocacy work for these victims are to be conducted) to close (11). Health-care services and police that are first-line responders are hardly available as they are overwhelmed too (48).

Due to the existing gender digital divide (62, 63), women in several nations, particularly those facing many forms of inequity, might be unable to access help services by cell phone or internet (owing to the lack of it). Even if these women have access to these means, they might be unable to use them because they are being closely watched by their perpetrators of violence. A Delhi-based NGO in India witnessed a 50% drop in calls on its helplines despite increased incidences of gender-based violence. This may have been due to the fear of getting discovered by their offenders (63, 64). As the use of online platforms has increased, women's rights experts and other bodies have reported an increase in varied forms of online violence against women, including bullying, stalking, sex-trolling, and sexual harassment (51, 53, 65).

The Ebola crisis with its resultant school closures showed that several forms of violence had worsened during the national health emergency, such as sexual exploitation and abuse of girls of reproductive age, child marriage, and trafficking (66, 67), and COVID-19 is following a comparable trend (68). The global cost of violence against women is bound to increase as gender-based violence increases during the pandemic and will sadly keep rising in the aftermath (7, 51).

### Health-Care Impacts of COVID-19

Epidemics limit access to the healthcare system particularly preventive and reproductive health care (11). Evidence from previous health crises reveals that obstetric care is particularly compromised (11). During a widespread health crisis, such as COVID-19, the unique health needs of women are more unlikely to be met as access to satisfactory health services, reproductive and maternal health care, essential medicines, and vaccines are undermined. The availability of maternal health care with sexual and reproductive health services and gender-based violence-linked services is crucial to the health, welfare, and rights of women. When resources are diverted from these provisions/services, increased maternal mortality and morbidity are the result (7).

The upsurge in cases of COVID-19 is seriously hurting both the wealthiest and most sophisticated health systems. With the ongoing pandemic, honest fears arise about the survival capability of less-developed countries (with frail health systems) (7). From previous national health crises, the diversion of funds from important but less urgent health services, such as maternal care and gender-based violence response, to focus on the health emergency at hand is the advantageous approach, yet important services need not be completely abandoned even at such critical times (8, 11). Conceding the reproductive and sexual health of girls and women during the ongoing pandemic implies many will likely experience pregnancy. According to Guttmacher Institute modeling estimates, a reduction by 10% in short- and long-term use of contraceptives could result in more than 15 million unintended pregnancies across 132 low- and middle-income countries (69).

### Limitations of the Study

The study was primarily based on published literature obtained from only three major databases. However, the non-systematic nature of the review represents a limitation in terms of the validity of the findings. Reports from unverifiable sources such as blog sites that were not documented in a verifiable source were not analyzed. The limited evidence for undeveloped/developing regions, or the general unawareness and even blackout regarding gender disparities and inequalities in COVID-19 crises were limitation factors in this study. Nevertheless, the strength of the study lies on the review of parameters that relate to women in the current COVID-19 health crisis. These included gender specificity, economic toll, pregnancy, gender-based violence, and the health-care impacts of COVID-19 on the health status of women around the globe.

## Conclusion

This article has reviewed some parameters as it relates to women in the current COVID-19 health crisis. Women are at a higher risk of infection with the COVID-19 virus due to their exposure as caregivers. COVID-19-infected pregnant women are at greater risk of other severe diseases including hospitalizations. This is most likely due to the physiological changes and exposure risks during antenatal care and childbirth. More women than men work in the informal sector of the economy, which was the worst hit by the lockdown directive that followed the outbreak of the pandemic. Many women became pregnant during the lockdown as a result of not being able to go to their places of work. Couples spent more time together, and this led to pregnancies for even some who did not plan on getting pregnant (9). The unpaid care burden for the sick, elderly, children, and adults locked down at home fell disproportionately on women. The gender-based violence increased across the globe as physical distancing measures were taken to limit the spread of COVID-19. The unique healthcare needs of women, which include sexual and reproductive health care, maternal health care (antenatal and postnatal care), essential medicines, immunization, and gender-based violence-linked services should not be downplayed during health emergencies. The approach by key players to tackle the impacts of the COVID-19 pandemic will be inefficient if it does not have a gendered stance considering the peculiar needs of women. Governments should therefore provide social security to ease their burden.

### Recommendations

Further research in the future is recommended to provide insight on how cultural and racial differences as well as other determinants of health (such as community, education, and the neighborhood) are impacting women during the COVID-19 pandemic. As useful data emerges with time, more investigations to understand the impact of COVID-19 pandemic on women's health across a broader geographic area (especially comparing more developed countries with those of the less developed countries) is imperative. Additional systematic studies to comprehend the overall effect of the COVID-19 pandemic on women's health is important to improve the wealth of scientific knowledge.

## Author Contributions

SO carried out the concept, design, and main interpretation of the study. VS, AC, SA, and UO conducted the electronic searches. VS drafted the manuscript and participated in the literature search and paper analysis. SO and VS critically reviewed and revised the manuscript for important intellectual content. UO participated in the proof editing. All authors made substantial, direct, and intellectual contributions to the work. The final version of the manuscript was read by all authors before giving their consent for its publication.

## Conflict of Interest

The authors declare that the research was conducted in the absence of any commercial or financial relationships that could be construed as a potential conflict of interest.
